# Capacity building of the Australian Aboriginal and Torres Strait Islander health researcher workforce: a narrative review

**DOI:** 10.1186/s12960-019-0344-x

**Published:** 2019-01-30

**Authors:** Shaun C. Ewen, Tess Ryan, Chris Platania-Phung

**Affiliations:** Melbourne Poche Centre for Indigenous Health, University of Melbourne, 141 Barry Street, Carlton, 3053 Australia

**Keywords:** Workforce, Research capacity building, Aboriginal and Torres Strait islander, Review, Health

## Abstract

**Background:**

This paper provides a narrative review that scopes and integrates the literature on the development and strengthening of the Australian Aboriginal and Torres Strait Islander health researcher workforce. The health *researcher* workforce is a critical, and oft overlooked, element in the health workforce, where the focus is usually on the clinical occupations and capabilities. Support and development of the Australian Aboriginal and Torres Strait Islander health researcher workforce is necessary to realise more effective health policies, a more robust wider health workforce, and evidence-led clinical care. This holds true internationally. It is critical to identify what approaches have resulted in increased numbers of Aboriginal and Torres Strait Islander people in health research, stronger local community partnerships with universities and industry, and research excellence and have contributed to evidence-led health workforce development strategies.

**Methods:**

The search was for peer-reviewed journal articles between 2000 and early 2018 on capacity building of the Aboriginal and Torres Strait Islander health researcher workforce. Databases searched were CINAHL (EBSCO), PubMed, PsychINFO, LIt.search, and Google Scholar, combined with manual searches of select journals and citations in the grey literature. A coding scheme was developed to scan research coverage of various dimensions of health research capacity building.

**Results:**

Twenty-four articles were identified. Eight focused on strengthening research capabilities of community members. A recurrent finding was the high research productivity of Aboriginal and Torres Strait Islander health researchers and strong interest in furthering research that makes a substantive contribution to community well-being. Action-based principles were derived from synthesis of the findings. Generally, research capacity building led to numerous gains in workforce development and improving health systems.

**Conclusions:**

There is a shortage of literature on health researcher workforce capacity building. National-level research on capacity building strategies is needed to support the continued success and sustainability of the Australian Aboriginal and Torres Strait Islander health researcher workforce. This research needs to build on the strengths of Aboriginal and Torres Strait Islander researchers. It also needs to identify clear and robust pathways to careers and stable employment in the health workforce, and health researcher workforce more specifically. This need is evident in all settler colonial nations (e.g. Canada, United States of America, New Zealand), and principles can be applied more broadly to other minoritised populations.

## Introduction

Building capacity of the Australian Aboriginal and Torres Strait Islander health researcher workforce creates a well-rounded and stronger working environment in Indigenous health [1] and disrupts research foci that have, historically and internationally, not been on Indigenous-led health priorities [2]. Research is fundamental to informing decisions on how to improve quality of health services [3], and local health workforces rely on research to back their requests for public health resourcing, such as staffing for sustained health care delivery [4]. The work that Indigenous health researchers do informs, guides, and critiques policy and influences how Aboriginal and Torres Strait Islander health policy is delivered [1]. Leadership by Indigenous researchers in accumulating evidence of the cultural inappropriateness of much health care service delivery, and the addressing of this core aspect of health care quality via policy reform [5, 6] is one of many examples of the important contribution that the research workforce makes to the performance of the wider health workforce.

The purpose of this paper is to provide a review that integrates literature from 2000 onwards, to inform research and policy directions on research capacity building (RCB) of the Aboriginal and Torres Strait Islander health researcher workforce, and to provide an evidence base that other minoritised populations internationally can draw from. Given that the Aboriginal and Torres Strait Islander population, which comprises 2.8% of the total [7] and experiences higher rates of ill-health and shorter life expectancy than other Australians [8], a significant expansion of the Aboriginal and Torres Strait Islander health researcher workforce is vital to better health outcomes [9].

A milestone in centring Aboriginal and Torres Strait Islander people in the health workforce were the Indigenous Research Agenda Reforms connected to the Cooperative Research Centre for Aboriginal and Tropical Health, between 1998 and 2002 [1, 10]. These reforms included training of more Aboriginal and Torres Strait Islanders in health research and introducing inter-organisational processes and mechanisms to embed more inclusive, ethical and impactful research [10]. In building on these earlier reforms, the Lowitja Institute [11] and others [12] have highlighted the need for a consolidated knowledge base of what RCB activities support the growing health workforce and what research approaches are effective in addressing health inequities for First Nations people in Australia and around the world [13–15]. Such a knowledge base would also include information about core agendas in respecting, meeting and securing Indigenous people’s rights, such as to the right to health, right to self-determination, the right to full employment, and right to high standard health services [16, 17].

### Background

Australian Aboriginal and Torres Strait Islander people have until recently been excluded from participation in the health knowledge economy [18]. The health research paradigm has historically been dominated by non-Indigenous researchers and policy-makers globally. This history includes practices that have devalued Indigenous peoples’ cultures, knowing and methods that are integral to realising health research premises, directions and modalities that are substantive, relevant, ethical and effectual [2, 9, 10]. Propelled by Indigenous leadership and extensive negotiating, the last two decades have seen significant movements in the research space [1, 19–21]:

- More Indigenous control of research processes with commitment to inclusion, reciprocity and research excellence,
- Shifts from *us and them* to collaborative research between Indigenous and non-Indigenous researchers and organisations,
- From a deficit-focused to strength-based gearing of education and research, and overturning inequities in education (participation and quality),
- Marked increases in Aboriginal and Torres Strait Islander people engaged in research, as indicated by the over twofold rise in doctoral students in the last 10 years,
- Increasing prominence of Aboriginal and Torres Strait Islander academics at high-level governance of universities,
- Prioritising of projects led or co-steered by Indigenous communities,
- Research going beyond observation-based studies of illness rates to cultural determinants of health,
- More recognition of how Aboriginal and Torres Strait Islander people have significantly advanced health research for the benefit of both Indigenous and non-Indigenous peoples,
- Ongoing significant under-representation in the health workforce (i.e. medicine), with targeted and successful strategies for improvement.

International literature (including Australian) highlights several central pillars which support health research workforce development [22–25]: RCB programmes, leadership, full pathways to careers, research opportunities for service-based health practitioners, strategic funding and recruitment [26]. In relation to RCB of the Aboriginal and Torres Strait Islander health researcher workforce, such themes have been underpinned by an emphasis on critical mass, vastly increased recognition of the value of Indigenous philosophies, knowledges, and methods in reshaping health research, and community-based participatory research.

A workshop run by the Lowitja Institute identified the need for a nationwide scoping review of progress in RCB [11], p. 5. The Lowitja Institute is Australia’s national research Institute for Aboriginal and Torres Strait Islander health research. The Lowitja Institute determined that stakeholders in RCB will be better positioned to support Aboriginal and Torres Strait Islander health researcher workforce strategic planning when previous research is analysed and understood as a whole. This includes the need to delineate the extent, types and findings of research on RCB for the two formal education sectors which are central pathways to workforce (Universities; Vocational Education and Training; VET) and the role, thus far, of community-based action research in RCB. Accordingly, the purpose of this work is to scope and synthesise (1) volume and proportion of articles devoted to RCB key areas (e.g. funding, mentoring), (2) research studies on RCB and (3) organisational and research training features, such as types of actions, that are associated with positive RCB outcomes.

## Methodology

### Inclusion criteria and search strategy

Sources were included in the review if they (1) were peer-reviewed journal articles, (2) had capacity building of the Australian Aboriginal and Torres Strait Islander health researcher workforce as a major focus, (3) were published between January 2000 and March 2018 and (4) were in English. The year 2000 marks the midway point of the Indigenous Research Reform Agenda to reconfigure and transform research, especially through centering of Aboriginal and Torres Strait Islander people in research [10]. To reiterate, a research need was identified to scope RCB initiatives, and research workforce changes since these reforms [11].

Searches were conducted from January to March of 2018. The searched databases were PubMed, PsychINFO, CINAHL (EBSCO), Lit.Search and Google Scholar. Titles, abstracts and key words (or subjects) were searched for the following terms in combination: “research capacity building” OR “research training” OR “research workforce” AND Aboriginal OR “Torres Strait Islander” OR Indigenous. Outlets exclusively by and/or on Indigenous peoples were manually searched: Aboriginal and Torres Strait Islander peoples (Australian Aboriginal Studies, Aboriginal and Islander Health Worker Journal, Australian Journal of Indigenous Education) and the International Journal of Indigenous Health. Via Google Scholar, we checked citations of articles directly about RCB. We also conducted a manual search of the citation lists of each new article that met the inclusion criteria, and undertook a search of reference lists in the grey literature. In addition, we scanned the websites of researchers in Australia that were known by the team to concentrate on Indigenous health, and those individual stakeholders identified through attendance lists to health RCB workshops, roundtables and other events reported in publicly available materials.

### Data extraction and analysis

We mapped the literature on generic fields such as author(s), region, research purpose, general study design, methods and findings. Various forms of RCB (e.g. mentoring) were also recorded. These codes provided the basis for comparing aspects of each paper. In order to identify research model features that may be connected with positive RCB outcomes, the most in-depth publications on discrete RCB programmes were analysed in detail, supplemented by a search for principles of capacity building in the remaining literature. The analysis of RCB programmes included attention to what publication authors highlighted as critical elements to success, as well as discerning consistencies across publications in features that corresponded with good outcomes (e.g. gaining employment after the research training). A working list of research model characteristics was derived out of the analyses.

## Findings

Twenty-four publications were identified [4, 27–49]. Table 1 describes the literature in terms of regional context, type of publication and aspects of RCB.

**Table 1:** Features of literature (authorship, regional scope, area of health research and coverage of attention to various aspects of research capacity building and workforce development)

First author and year	Exclusively on RCB and/or health research workforce	Aboriginal and Torres Strait Islander authors (LA: Lead author)	Scale	Area of health research	Research capacity building					Aspects of research capacity building								Primary research: experiences and views of researchers		Outcomes measurement
					Students	Academics	Health care professionals	Community members	Training non-Indigenous health researchers	Discrete programme	Pathways/Transitions	Mentoring	Partnership building	Interaction of research cultures	Research governance	Health research leadership	Funding	Qualitative	Quantitative	
Tsey, 2001 [43]		✓(1) LA	NT	General											✓					
Bailey, 2006 [30]	✓		QLD	Community health			✓			✓										
Brands, 2006 [46]		✓ (1)	National	General			✓	✓	✓				✓		✓	✓				
Foster, 2006 [29]	✓	✓ (5) LA	NT	Alcohol											✓					
Street, 2007 [42]		✓ (1)	National	General		✓		✓					✓		✓		✓	✓		
Rumbold, 2008 [48]			National	General															✓	
de la Barra, 2009a [35]		✓ (1)	National	General									✓			✓	✓	✓		
de la Barra, 2009b [36]		✓ (1)	National	General		✓											✓			✓
Mayo , 2009 [28]			QLD	General				✓						✓	✓			✓		
Mooney-Somers, 2009 [47]			QLD, NSW, WA	Community health				✓					✓							
Saunders, 2010 [41]		✓(2) LA	Nth QLD	Nursing	✓									✓				✓		
Clapham, 2011 [34]		✓ (1) LA	National	Public health, services				✓							✓	✓				
Guthrie, 2011/2 [39]	✓	✓ (2) LA	National	Epidemiology	✓					✓								✓		✓
Kelly, 2012 [40]	✓	✓	QLD, NSW, WA	Public health				✓		✓			✓					✓		
Elston, 2013 [37]	✓	✓ (2) LA	QLD	Public health, nursing, social science, primary health, community health						✓						✓				✓
Bainbridge, 2016 [54]	✓	✓ (1) LA	National	General		✓					✓	✓	✓			✓		✓		
Bainbridge, 2016 [31]	✓	✓ (11) LA	National	General		✓						✓			✓	✓	✓	✓		
Davis, 2016 [49]			National	Epidemiology	✓			✓		✓		✓	✓				✓			
Gray, 2016 [38]			National	General														✓		
Young, 2016 [44]		✓ (2)	NSW	General			✓						✓					✓		
Hickey, 2017 [45]	✓	✓ (3)	SE QLD	Midwifery			✓	✓			✓	✓	✓					✓		
Cartwright, 2018 [33]			QLD	Alcohol and other drugs/health services			✓													
McPhail-Bell, 2018 [27]	✓	✓	National	Primary health		✓	✓		✓	✓			✓		✓	✓				
Nichols, 2018 [4]	✓		Nth QLD	Health promotion			✓			✓		✓						✓	✓	✓

### Layout of literature

Table 2 provides a stocktake of this literature, showing that the highest percentages were for RCB in health generally or multiple health fields, RCB of community members, and partnership building, and qualitative studies of researcher trainee experiences. Half of the literature considered national-level RCB. The other half demonstrated local or regional approaches. Furthermore, only 10 of the 24 articles had RCB of Aboriginal and Torres Strait Islander health researchers as the sole focus. Table 3 presents more detail on the purpose of each publication, research context and design. We now turn to a brief synthesis of the literature.

**Table 2:** Proportion of literature by aspect of RCB

Area of RCB	Number of articles	Percentage
Exclusively on RCB and/or health research workforce	10	41.7
Aboriginal and Torres Strait Islander authorship	16	66.7
Scale		
National	12	50.0
Australian Capital Territory	0	0.0
Northern Territory	2	8.3
Queensland	9	37.5
New South Wales	3	12.5
Western Australia	2	8.3
Victoria	0	0.0
Tasmania	0	0.0
Area of health research		
General/multiple	12	50.0
Epidemiology	2	8.3
Nursing	2	8.3
Midwifery	1	4.2
Alcohol and other drugs	2	8.3
Public health	3	12.5
Research capacity building		
Students	3	12.5
Academics	5	20.8
Health care workers/professionals	7	29.2
Community members	8	33.3
Training non-Indigenous health researchers	2	8.3
Aspects of RCB		
Discrete programmes	7	29.2
Pathways/transitions	2	8.3
Mentoring	5	20.8
Partnership building	10	41.7
Interaction of research cultures	2	8.3
Research governance	8	33.3
Health research leadership	7	29.2
Funding	5	20.8
Primary research: experiences of researcher trainees		
Qualitative	12	50.0
Quantitative	2	8.3
Outcomes measurement	4	16.7

**Table 3:** Research literature on the Aboriginal and Torres Strait Islander Health Researcher Workforce

Author(s)	Purpose	Research context	Research training programme features	Design and sample	Funding
Tsey, 2001 [43]	To introduce the formation of the Cooperative Research Centre for Aboriginal and Tropical Health and the series of consultations contributing to RCB strategies under the Indigenous Education and Health Program	Research agenda development in the Northern Territory, shaped by partners across health research organisations, health care services, education providers and government	N/A	N/A	
Bailey et al., 2006 [30]	Describe development and structure of a new VET-accredited research course to facilitate community research capacity via training of health workers	Research training of Aboriginal and Torres Strait Islander health workers in community settings	Six RCB course modules: ‘existing services’, ‘identifying need’, ‘program development’, ‘service improvement’, ‘outcomes’, and ‘evaluation measures’	N/A	Department of Health and Ageing
Brands, Gooda, 2006 [43]	Outline development of the Cooperative Research Centre for Aboriginal Health (CRCAH) and impacts on governance, direction and modalities of health research	Establishment of CRCAH and strategies for increasing relevance and health impact of research (inc. research capacity building)	Brief mention of traineeships (p. 28)	N/A	
Foster et al., 2006 [29]	Demonstrate establishment of a local Aboriginal researcher network and its effectiveness and other benefits	Development of Council-based and Aboriginal-led research (council staff residents), beginning with a survey on local views on a substance use restriction intervention	Workshop (1 week) conducted by two health researchers	N/A	Central Australian Division of Primary Health Care and the Centre for Remote Health
Street, Baum, Anderson, 2007 [42]	Attain views of researchers within organisations associated with the CRCAH on how research funding is organised (e.g. whether should be based on competition or collaboration)	Exploring views on the approach, processes and tenor of research funding by the CRCAH	N/A	Semi-structured qualitative interviews, with six experienced Aboriginal researchers, all in organisations connected with the CRCAH	
Rumbold et al., 2008 [48]	Profile the Aboriginal and Torres Strait Islander research workforce and gain health researcher views	Part of a research programme called Capacity-building in Indigenous Policy-relevant Health Research	Not on a discrete programme	Survey, cross-sectional, *n* = 373, 32 (9%) Aboriginal and Torres Strait Islander (38% employed at university and 22% government)	NHMRC
de la Barra et al., 2009 [35]	Compare effectiveness in advancing research capacity of two NHMRC schemes for the period 1996 to 2006: People Support and Capacity Building in Population Health	Government research funding at national level	Funding models: People Support and Capacity Building	Analysis of NHMRC data	
de la Barra, Redman, Eades, 2009 [36]	To explore, in depth, changes within the NHMRC, especially the growing influence of Aboriginal and Torres Strait Islanders in this organisation	Internal structures and policies of NHMRC and commitment to Indigenous health research	Research Projects funded by NHMRC People Support or Capacity Building in Population Health NHMRC policy change process and key relationships	Case study: projects funded by either People Support or Capacity Building in Population Health Interviews 7 leaders influencing NHMRC policy changes	Sydney University postgraduate scholarship
Mayo et al., 2009 [28]	Describe and reflect on long-term community partnerships with university researchers in establishment and management of health programmes (e.g. challenges and benefits for collaborators)	Workshop-facilitated collaboration between community-based and university-based researchers within a participatory research framework connected with health programmes (Family Well-Being Programme and Indigenous Men’s Support) at Gurriny Yealamucka health service	Learning of research during community-controlled health programme implementation and evaluation	Qualitative interviews (*n* = 5) and analysis of workshop transcripts	Not stated
Mooney-Somers, Maher, 2009 [47]	Case study of action research with built-in RCB of young community members and cross-organisation partnership building: Indigenous Resiliency Community-Based Participatory Research Project	Protection of young people from sexually transmissible infections and blood-borne viruses	Training of peer researchers from the community via workshops. Peer researchers co-conduct interviews	Case study of the research project	International Collaboration in Indigenous Health Research Program
Saunders, West, Usher, 2010 [41]	Share student and supervisor stories of learning about and drawing on Indigenist research methodologies	At James Cook University, two Indigenous university students and their non-Indigenous supervisor negotiate (individually and jointly) the intersection of Indigenous research methodologies (e.g. Rigney) and Western research	N/A	N/A	NHMRC Research Capacity Building
Clapham, 2011 [34]	Reflect on the benefits of Indigenous leadership in health research, avenues of leadership development, and challenges	Several prevention research studies (esp. injuries) in NSW	N/A	N/A	Not stated
Guthrie et al., 2011–2012 [39]	Evaluative reflection on effectiveness of the Master of Applied Epidemiology (MAE) in RCB	MAE at ANU’s National Centre for Epidemiology and Population Health (funded by Department of Health and Ageing)	Coursework degree (3 months, over 2 years) with an intensive field placement in applied epidemiology (21 months)	Interviews and surveys, *n* = 13	Not stated
Kelly et al., 2012 [40]	Unpack research experiences and types of influences of research training and collaboration on research engagement and potential effects on the researcher’s planning	Multi-disciplinary, community-based research on ‘impacts of influenza’ in rural/remote Three states (specific sites: Tamworth, Inverell, Palm Island, Innisfail, Torres Strait, Bidyadanga) Indigenous and non-Indigenous researcher partnerships	Qualitative research training workshops (18 days): data collection/analysis and writing; partnering with experienced researchers Education principles: ‘learn by doing’, ‘two-ways learning’ Workshop attendees with different work backgrounds; diverse types of recruitment into the workshop	Interviews: telephone/email, *n* = 8	NHMRC
Elston et al., 2013 [37]	Reflect on the challenges and successes of a RCB programme: Building Indigenous Research Capacity (BIRC) Collective	James Cook University	Programme positioned “within a framework of Indigenous leadership, ownership and engagement” (p. 11) Regular mentoring Workshops (5 days, 2 times a year for each of 5 years): gathering/sharing and research skills Writing retreats from 2009	Mixed methods, Indigenous knowledge centred Three components: (1) an evaluator from outside the Project, interviews and soliciting writing on reflections on the Project experience; (2) two members of the Project jointly doing a thematic analysis of narratives from the Project Community Report released in Year 5; (3) project-lifespan frequencies of outputs, such as new grants and publications	Capacity Building in Population Research Grant (NHMRC)
Bainbridge, 2016 [54]	National Indigenous Research Knowledges Network (NIRAKIN) support of Aboriginal academics	Research professional and leadership development over 5 years of involvement in NIRAKIN, esp. health node	Mentoring and team-based research, including leadership development Solidarity with women (p. 8) Extension to funding	Auto-ethnography (journaling and thematic analysis)	Not stated
Bainbridge et al., 2016 [31]	Reflect on co-development of health research linkages within the NIRAKIN	First 4 years of collaboration of university-based Aboriginal and Torres Strait Islander academics via the Health and Wellbeing node of NIRAKIN	Culturally safe online meetings Resource-supported partnership building	Collaborative auto-ethnography Emic, narrative 11 Aboriginal and Torres Strait Islander researchers, and one non-Indigenous researcher	Not stated
Davis et al., 2016 [49]	Evaluate progress after revisions to the MAE Program, including a new funding framework, from 2012	Formal degree classification changed to a research degree. Organisations offering field placements now contribute to the funding, alongside the internal funding by the National Centre for Epidemiology and Population Health at ANU	See Guthrie et al. above	Internal document analysis	Not stated
Gray, Oprescu, 2016 [38]	Review Australian literature on the place of non-Indigenous researchers in research focused on Indigenous health	Current and future roles of non-Indigenous researchers	N/A	Literature review Thematic analysis	Not stated
Young et al., 2016 [44]	Attain views of Aboriginal and Community-Controlled Health Service (ACCHS)-based health professionals on data (e.g. access)	ACCHS perspectives on data derived from research and/or clinical purposes—to inform local RCB and research partnerships with other health organisations.	N/A	Semi-structured interviews: 35 health professionals (urban and regional ACCHSs) Thematic analysis	NHMRC (Study of Environment on Aboriginal Resilience and Child Health)
Cartwright et al., 2018 [33]	Case study of electronic survey methods of collecting evaluation data as a means of RCB within community-based Indigenous health organisations	Queensland Aboriginal and Islander Health Council evaluation of their own organisational performance: culturally safe use of wireless platforms for self-report feedback by participants on workshops on alcohol and other drugs services (AOD-our-way and Crystal Clear workshops)	N/A	Case study, qualitative	Queensland Health and Australian Government
McPhail-Bell et al., 2018 [27]	To introduce an Indigenous research capacity building model for improving quality of health care provision	Embedding, in primary health care, a research-oriented and Indigenous led continuous quality improvement system	Setting policy and network structures for an “all teach, all learn” base for research capacity building.	N/A	NHMRC
Nichols et al., 2018 [4]	Demonstrate increasing capacity of health promotion staff to conduct and manage research evaluation of local programmes	Research learning of a four-member health promotion team within the Apunipima ACCHS and subsequent improvements to on-site programme evaluation systems	Workshops and ongoing sessions of mentoring, finishing with a conference presentation. Trainers and mentors within the ACCHS and from James Cook University	Surveys (*n* = 33 after workshops; *n* = 27 after mentoring meetings)	Not stated

### Strengthening the health workforce

Several RCB initiatives were taking place within Councils [29, 33] and other community health services [4], as well as university training programmes that support public health strengthening [49]. Across these cases of RCB, immediate gains in workforce development and improving health systems were found, including:

- Enhanced service evaluation skills of health care staff and broader programme assessment capacity of health organisations [4, 33],
- After research training and experiences, envisioning a career in the health care workforce [45] and higher employability [37, 49],
- New arrangements for health care providers to openly discuss and reshape the purposes and uses of research data [44, 46],
- As an outcome of research that took place during a placement: changes to legislation, paving the way for health data linkage [39].

Overall, Aboriginal and Torres Strait Islander peoples conducting research do vital work in improving infrastructure of the broader health workforce, such as in the frontline service delivery environment.

### Research capacity building funded by the National Medical Health and Research Council

Research in Australia is primarily funded by the Australian Research Council (ARC) and the National Medical Health and Research Council (NHMRC), two Australian government entities. Aboriginal and Torres Strait Islander people are increasingly influencing the strategic funding of research and the setting of minimal funding targets for Indigenous health-related research [50]. In addition, focusing on and tracking the proportion of Indigenous-led research projects and programmes has become more prominent. For instance, it was noted that 50 of the 546 Indigenous health research grants funded by the NHMRC were headed by an Aboriginal and/or Torres Strait Islander researcher between 2010 and 2016 [51]. Similar and related strategies can be found in Canadian and New Zealand health research councils [52, 53].

Literature on the effectiveness of national research funding strategies to support Aboriginal and Torres Strait Islander capacity building showed some success. De la Barra et al. [36] compared the effectiveness in advancing research capacity of two NHMRC schemes for 1996 to 2006—(1) People Support and (2) Capacity Building. “People Support” was a scholarships and fellowships scheme, providing salaries to individual researchers, including early and mid-career academics. “Capacity Building” was a more targeted scheme in that it focused on health services and public health, and within these fields, on team research projects and programmes whereby an early career researcher was mentored by either the chief investigator and/or senior-level health researcher. It was found that “less than 10% of expenditure on People Support for Indigenous health was allocated to researchers who self-identified as Indigenous” ($1.1million, 27 Aboriginal and Torres Strait Islander researchers) [36], p. 30. Over 5 years since inception, the Capacity Building Grant had supported 36 Aboriginal and Torres Strait Islander researchers, well over double that of People Support (*n* = 14). Suggested reasons for more support of Aboriginal and Torres Strait Islander researchers by Capacity Building Grants included longer funding durations, and utilisation of a “multidisciplinary team mentoring model” [36], p. 31. Overall, RCB seems to be better supported by the grant scheme that secured collective capacity building, combined with a longer-term outlook.

Elston et al. [37] reflected on the challenges and successes of a RCB programme, funded over 5 years by the Capacity Building in Population Research Grant (NHMRC), with the research group called the Building Indigenous Research Capacity (BIRC) Collective at James Cook University. Factors deemed to have influenced the individual and collective achievements were as follows: “*Space – IK* [Indigenous Knowledge] *systems, Indigenous Ownership, Leadership and Respect, Identity, and working at the Cultural Interface*.” (authors’ italics, p. 11). Challenges were “cultural sensitivities, conflicting priorities and competing agendas (because of the vast differences amongst and within Indigenous Australia)…the varying skills, experiences and professional backgrounds of the Indigenous participants” (p. 8). From before BIRC to after, Indigenous researcher outputs went from 36 publications to 131, including an over fivefold increase in conference presentations and almost threefold increase in reports. BIRC members voiced concerns about sustainability, such as a lot of work-in-progress writing for submission to peer-reviewed journals at the end of the programme. The authors pointed out the need for more investment in place (e.g. prolonged funding) to ensure opportunities to further disseminate research and, as part of this, capitalise on the new capacities built during the programme.

### Other discrete university-based programmes that were successful in research capacity building

The Master of Applied Epidemiology (MAE) Program started in 1991, within Australia National University’s (ANUs) National Centre for Epidemiology and Population Health. It was a 2-year coursework degree, consisting of a 3-month intensive coursework and an intensive field placement in applied epidemiology for the remainder of the degree (21-month duration). The field placement would be either Australian based or international. The MAE has played a role in the strengthening of public health workforces and performance internationally. This is especially the case in relation to the establishment and improvement of communicable disease surveillance and response systems [49].

Guthrie et al. [39] reported the findings of an evaluation of the MAE Program, which was found to have “contributed greatly towards their [Aboriginal and Torres Strait Islander] professional development” (p. e106). MAE Aboriginal and Torres Strait Islander scholars had produced over 70 publications as well as over 100 conference presentations. Of the Aboriginal and Torres Strait Islander graduates, as of 2012, five had completed doctoral studies and seven were enrolled in a doctorate (p. 106). At the time of publication, there have been 187 MAE graduates, of whom 29 were Aboriginal and/or Torres Strait Islanders [49]. In sum, MAE represents a successful RCB initiative in terms of providing international experiences and networks, grounded expertise in epidemiology and its application in real-world settings, high research dissemination (e.g. publications) and excellent post-programme educational outcomes [39, 49].

### Research capacity building within participatory action research

In a case study report by Hickey et al. [45], two Aboriginal and Torres Strait Islander women shared their experiences of entering community-based health research for the first time and becoming research assistants involved in recruitment and interview-type data collection, the mentoring process, and the growth of research reflexivity and meaningful relationships with the research participants. After the research project, both sought to become professionals in the Australian health care system: one midwifery, the other (child) nursing.

### Viewpoints and experiences of Aboriginal and Torres Strait Islander students and current health researchers

There were 12 reports on Aboriginal and Torres Strait Islander emerging and senior researcher viewpoints and experiences [4, 28, 31, 37–41, 44, 45, 48, 54]. Rumbold et al. [48] compared Aboriginal and Torres Strait Islander health researcher views to other health researchers. This was based on 373 survey respondents of whom 32 (9%) were Aboriginal and Torres Strait Islander (38% employed at a university, and 22% in government). For Indigenous health researchers, in response to the question “factors that make Indigenous health research activities attractive”, 62% indicated “important area/national priority”, 72% “opportunity to contribute to social justice”, 69% “opportunity to make a difference”, and 66% “opportunity to work with Indigenous people and communities.” (p. 15). There was a statistically significant higher proportion of Aboriginal and Torres Strait Islander researchers (cf. non-Indigenous researchers) who indicated that the “factors that make Indigenous health research unattractive” were “not enough institutional support”, “lack of critical mass”, and “not enough mentors”.

### Framework characteristics and successful research capacity building

Table 4 outlines what appear to be critical factors to effectiveness in RCB, based on our analysis of the literature. Of primacy are research training tenets at the top of the table in relation to embracing the leadership and other strengths of Aboriginal and Torres Strait Islanders. Furthermore, the characteristics of effective RCB are action-focused, oriented to relationship building, provision of opportunities for learning and co-construction of research agendas, sustained funding and enduring embracement by the wider organisation. Also of note, in terms of exemplifying the effective characteristics in Table 4, the former BIRC programme at James Cook University [37] was one of the most comprehensive RCB approaches.

**Table 4:** Research model characteristics

Organisational features	
• Privilege Indigenous worldviews, identities, experiences, knowledges, research, and pedagogical philosophies and methods, including inter-cultural workings [37, 41].	
• Recognise, value, and invest in Aboriginal and Torres Strait Islander health researchers *as* Indigenous [37, 41].	
• Deliver excellence-based research training strategies that are responsive to the strong desire of Aboriginal and Torres Strait Islander research trainees for high quality, ethical, actionable and impactful health research [37, 39, 40].	
• Sole or co-lead and manage by Aboriginal and Torres Strait Islander academics of research programme development, and implementation [37, 54].	
• Prioritise programme-level research (not investigator- or single study-driven), wedded to a long-term vision (including critical mass and outcomes-based research) [46].	
• Build (inter-generational) cohorts of Aboriginal and Torres Strait Islander health researchers [37, 54].	
• Orientate the programme to close partnerships with Indigenous communities, Elder shaping of research directions and Indigenous expertise [37, 40, 54].	
• Secure and sustain funding of the RCB model [36, 37, 39, 54].	
• Gain and retain support at the executive level of the institution [39].	
• Commit to organisational policy for research and training that factors in necessary time and flexibility for fortifying relationships [37, 40, 42].	
• Provide clear and viable post-completion pathways into health research careers and leadership positions [35, 49].	
• Network strategically as an organisation [39, 40]. For instance, install mechanisms to optimise trainee network spread and outreach: within-cohort, cross-institution, cross-country and international.	
• Be open to, and commit to navigating complex discipline inter-cultural values and priorities. Be cognizant of shared values (respect, integrity, responsibility, reciprocity) [37, 41].	
• To monitor and review RCB approaches, deploy mechanisms to attain data on outcomes and progress (e.g. feedback on training, post-completion employment, publications) [37, 39].	
Research training	
• Support trainees through structures and mechanisms responsive to needs (social, cultural, emotional, financial) [37, 41, 54].	
• Deliver support infrastructure that is attuned to the diversity of expertise, entry pathways, lived experiences, community/familial commitments, aspirations and mobilities of trainees [37, 40].	
• Secure trainee access to experienced supervisors and mentors (Indigenous and non-Indigenous) [37].	
• Support supervisors and mentors, including training of non-Indigenous supervisors in cultural competence and cultural safety [9].	
• Establish a diverse composition of research programme members, such as by discipline, level of research experience and specialist expertise (e.g. on social determinants of health, knowledge translation, services planning and evaluation) [36, 37].	
• Ensure a sustained set of relationship-building-focused and learning-focused meeting structures (courses, seminar series, workshops, retreats, lectures, reading groups) [37, 39, 54].	
• Deliver research training across the spectrum of research skill sets (e.g. writing, research plans, conference presentations, grant applications, project management) [37].	
• Provide spaces for welcoming and collaborative in-person engagement on a regular basis, including meetings exclusively between Indigenous peoples [37, 54].	
• Provision for ample opportunities for early and later career researchers to inter-mingle and join new research projects [37].	

## Discussion

This review identifies a steadily building body of literature related to RCB of the Aboriginal and Torres Strait Islander health researcher workforce [20]. It indicates that there are several concurrent developments in RCB, including national-level endeavours by Aboriginal and Torres Strait Islander-led structures (the Lowitja Institute and Cooperative Research Centres [46], National Indigenous Research and Knowledges Network [31]) and the NHMRC [36]. A standout finding was that investment (albeit, minimal and short-term) was followed by heightened success in research activity and outcomes, including higher levels of academic output, more frequent post-programme entry into further health research, new research partnerships and inter-disciplinary learning [37, 39]. Also recurrent is the notion that what may be integral to RCB success are strong relationships and devotion of time for partnership building [35, 37, 41]. Funding arrangements overlooked the time necessary to build quality relationships [42].

The literature reviewed raises several ways that capacity building of the research workforce facilitates capacities of the wider health workforce to better serve Aboriginal and Torres Strait Islander people. There were examples of newly research-trained community members deciding to enrol in university degrees to become health professionals [45]. As described in Nichols et al. [4], research learning can have immediate flow on effects in the capacity of Aboriginal and Torres Strait Islander health professionals in the primary care sector to more effectively demonstrate the value of their health programme approach to the local community, senior personnel in their service organisation and external funders of services. As a further example, closer relationships of local Aboriginal and Torres Strait Islander communities with health care providers are enabled by Aboriginal and Torres Strait Islander-led research on health-related matters [29]. Overall, Indigenous peoples conducting research do vital work in improving infrastructure of the broader health workforce, such as in frontline service delivery environments.

This paper supplements a review of literature on Aboriginal and Torres Strait Islander research students across all areas of university-based postgraduate education [20], by focusing on research training in the health fields, and within this scope, expanding to post-degree research careers, and RCB outside of universities. Similar to that review, it was found that there was a shortage of in-depth qualitative research on trainee experiences, minimal inquiry into quality of research training and the need for a more global outlook, such as re-positioning within the evolving collective agendas of First Nations researchers.

Through the current integration of literature, it became clear that there are numerous gaps in knowledge that need to be filled in order to be better placed to inform decisions in workforce development. In order to guide the role of research in workforce planning, Table 5 outlines research directions to advance knowledge in several areas pertinent to RCB. The majority of these directions reflect a need to detail and clarify how the education systems (VET, universities), Indigenous health research workforce and wider health workforce inter-lock. Accordingly, research progress in these areas would be valuable in determining how the health workforce may be most effectively strengthened.

**Table 5:** Overview of research tasks to address gaps in knowledge

Area of capacity building	Activities to either further detail or remedy research gaps
Pathways into health research training, work transitions and attraction to research careers in health	-Pinpoint organisational and life factors enabling and hampering health research interests. -Chart transitions and pathways to research training and employment in health research (e.g. medicine, midwifery, occupational therapy, dentistry, nursing). -Identify sector location and number of Aboriginal and Torres Strait Islander people considering a career in health research. -Map out recruitment strategies, such as attracting high performing emerging researchers to a career in the health sector.
Community-based health care professionals	-Identify the availability of opportunities for research learning. -Document systems of RCB driven by Aboriginal Community Controlled Health Organisations and health care practitioners. -Explore how participatory/action research projects grow research capacity, including stakeholder viewpoints on the benefits.
Employment conditions	-Ascertain the employment conditions and level of job security of Aboriginal and Torres Strait Islander health researchers. -Determine whether employment arrangements for health professionals provide incentives and resources for training and engagement in health research, and if so, how, and the outcomes.
Organisational loci of direct commitment to RCB	-Grasp which organisations in the health care and education sectors commit to Aboriginal and Torres Strait Islander health RCB in policy and in actual practice (e.g. health professional associations). -Clarify what capacity building activities are taking place in medical and health research institutes outside of universities (e.g. internship programmes). -Pinpoint what organisational structures support continued learning opportunities in research for current health professionals who may not have a research background. -Conduct case studies of programmes focused on health research leadership.
VET sector	-Increase visibility of research training in VET (including research components of health courses). -Recognise types and frequencies of health research roles and employment after completion of VET. -Establish a data base on VET and university bridging and transition pathways in health research training.
Discrete RCB models	-Discern the quality of relationships and research training in RCB programmes (e.g. evaluating, revising, extending Table 4). -Find out the views of Aboriginal and Torres Strait Islander trainees on congruence of pedagogies with research aspirations.
Funding	-Research on capacity building outcomes of ARC funded research, comparable to inquiry of the NHMRC. -Pin down the contribution of State and Territory-level government health departments and non-university medical and health research institutes to RCB. -Evaluate the progress of funding schemes in meeting the RCB goals of Aboriginal and Torres Strait Islander researchers and communities?
International RCB	-Better understand the antecedents, experiences and outcomes of Aboriginal and Torres Strait Islander students and health researchers engaged in research training overseas. -Inquire into RCB experiences and outcomes of First Nations researcher meetings. -Assemble lessons from RCB models focused on other minoritised peoples in Australia and other countries.

To bolster the RCB evidence base on best practice at a national level, of significant value would be primary research on the experiences and views of trainees (similar to those for health profession-specific RCB, e.g. see [55]) and the prospective evaluation of RCB frameworks. Additionally, research should inform debate on how to better connect RCB and workforce development, so that institutions provide research employment that gels with the trajectories of emerging researchers.

In turning to the global level, the research model characteristics that we have identified as associated with the most effective capacity building (Table 4) should be considered in the planning and design of research learning systems in other countries. The list of characteristics also provides a basis for international research on how extendable they are to capacity building in other health research contexts. Lessons garnered from this research, such as investing in the ongoing capacity development of Indigenous researchers and cohort-driven environments (as outlined in Table 4), can greatly assist the Indigenous health workforce internationally. Countries such as Canada and the United States can benefit from this review as part of further driving workforce development. This includes embedding and acknowledging the contribution that the Indigenous health workforce makes in improving health outcomes for all Indigenous populations.

This review provides an incomplete picture of the frequency and diversity of RCB. While the so-called grey literature was beyond the scope, the current searches unearthed sources documenting RCB initiatives, and trainee reflections, e.g. [56]. A review of the grey literature is needed to provide a more representative snapshot of strategies in RCB, and workforce development.

## Conclusion

The continued growth and development of Aboriginal and Torres Strait Islander health researchers leading health research is a critical element of shaping the health research agenda, and in turn, the well-being of Aboriginal and Torres Strait Islander people. Robustness of the Indigenous health workforce is well served by facilitated growth in the number of Indigenous health researchers. This review identified a paucity of evidence-based literature on RCB throughout the areas pertinent to research workforce development. We call for a redoubling of efforts to upscale and accelerate programmatic research on RCB strategies centred on the strengths of Aboriginal and Torres Strait Islander people, and that prioritise dialogue between local communities, health care and research organisations, and all tiers of government, with due attention to systemic and structural issues. Such research directions may also be applicable to other minoritised peoples in Australia and globally.

## Abbreviations

ANU: Australian National University
ARC: Australian Research Council
BIRC: Building Indigenous Research Capacity
MAE: Master of Applied Epidemiology
NHMRC: National Health and Medical Research Council
RCB: Research capacity building
VET: Vocational Education and Training
